# (Butane-1,2,3,4-tetraol-κ^3^ *O* ^1^,*O* ^2^,*O* ^3^)(ethanol-κ*O*)tris­(nitrato-κ^2^ *O*,*O*′)holmium(III)

**DOI:** 10.1107/S160053681300305X

**Published:** 2013-02-20

**Authors:** Xiao-Hui Hua, Jun-Hui Xue, Li-Min Yang, Yi-Zhuang Xu, Jin-Guang Wu

**Affiliations:** a Beijing National Laboratory for Molecular Sciences, The State Key Laboratory of Rare Earth Materials Chemistry and Applications, College of Chemistry and Molecular Engineering, Peking University, Beijing, People’s Republic of China; b Chemical Engineering College, Inner Mongolia University of Technology, People’s Republic of China; c State Key Laboratory of Nuclear Physics and Technology, Institute of Heavy Ion Physics, School of Physics, Peking University, Beijing, People’s Republic of China

## Abstract

In the title Ho^III^–erythritol complex, [Ho(NO_3_)_3_(C_4_H_10_O_4_)(C_2_H_5_OH)], the Ho^III^ cation is chelated by a tridentate erythritol ligand and three bidentate nitrate anions. An ethanol mol­ecule further coordinates the Ho^III^ cation, completing the irregular O_10_ coordination geometry. In the crystal, an extensive O—H⋯O hydrogen-bond network links the mol­ecules into a three-dimensional supra­molecular structure.

## Related literature

For crystal structures of related lanthanide nitrate–erythritol complexes, see: Gyurcsik & Nagy (2000 ▶); Yang *et al.* (2003 ▶, 2004 ▶, 2012 ▶).
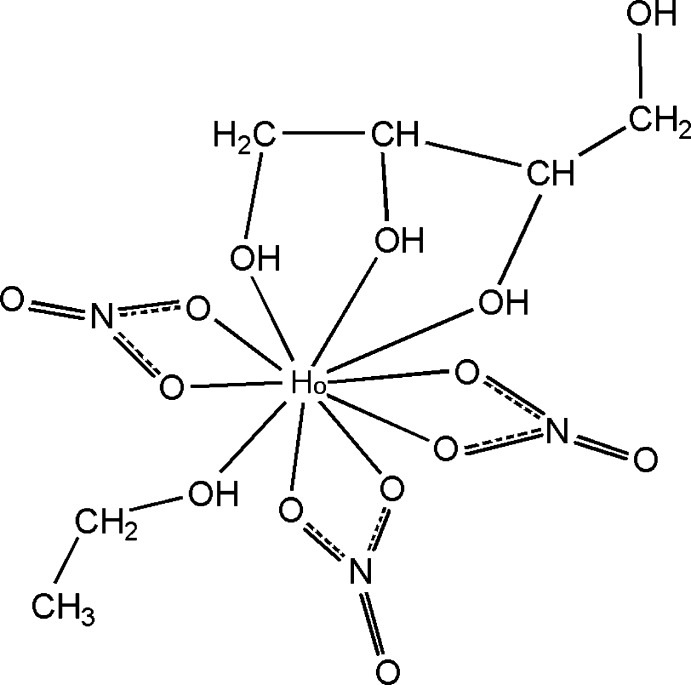


## Experimental

### 

#### Crystal data

[Ho(NO_3_)_3_(C_4_H_10_O_4_)(C_2_H_6_O)]
*M* *_r_* = 519.15
Monoclinic, 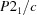

*a* = 7.7501 (16) Å
*b* = 12.783 (3) Å
*c* = 15.164 (3) Å
β = 100.35 (3)°
*V* = 1477.8 (5) Å^3^
*Z* = 4
Mo *K*α radiation
μ = 5.44 mm^−1^
*T* = 173 K
0.26 × 0.19 × 0.19 mm

#### Data collection

Rigaku Saturn724+ CCD diffractometer
Absorption correction: multi-scan (*CrystalClear*; Rigaku, 2007 ▶) *T*_min_ = 0.25, *T*_max_ = 0.36
10146 measured reflections
3376 independent reflections
3198 reflections with *I* > 2σ(*I*)
*R*_int_ = 0.038

#### Refinement

*R*[*F* ^2^ > 2σ(*F* ^2^)] = 0.029
*wR*(*F* ^2^) = 0.061
*S* = 1.19
3376 reflections
218 parameters
H-atom parameters constrained
Δρ_max_ = 1.11 e Å^−3^
Δρ_min_ = −0.83 e Å^−3^

### 

Data collection: *CrystalClear* (Rigaku, 2007 ▶); cell refinement: *CrystalClear*; data reduction: *CrystalClear*; program(s) used to solve structure: *SHELXTL* (Sheldrick, 2008 ▶); program(s) used to refine structure: *SHELXTL*; molecular graphics: *SHELXTL*; software used to prepare material for publication: *SHELXTL*.

## Supplementary Material

Crystal structure: contains datablock(s) global, I. DOI: 10.1107/S160053681300305X/xu5656sup1.cif

Supplementary material file. DOI: 10.1107/S160053681300305X/xu5656Isup2.cdx

Structure factors: contains datablock(s) I. DOI: 10.1107/S160053681300305X/xu5656Isup3.hkl

Additional supplementary materials:  crystallographic information; 3D view; checkCIF report

## Figures and Tables

**Table 1:** Selected bond lengths (Å)

Ho1—O1	2.367 (3)
Ho1—O2	2.373 (3)
Ho1—O3	2.473 (3)
Ho1—O5	2.364 (3)
Ho1—O6	2.443 (3)
Ho1—O7	2.444 (3)
Ho1—O9	2.449 (3)
Ho1—O10	2.497 (3)
Ho1—O12	2.590 (3)
Ho1—O13	2.445 (3)

**Table 2:** Hydrogen-bond geometry (Å, °)

*D*—H⋯*A*	*D*—H	H⋯*A*	*D*⋯*A*	*D*—H⋯*A*
O1—H1⋯O4^i^	0.84	1.86	2.665 (4)	161
O2—H2⋯O12^ii^	0.84	1.99	2.794 (4)	159
O3—H3⋯O10^iii^	0.84	2.08	2.914 (4)	177
O4—H4⋯O14^iv^	0.84	2.12	2.894 (4)	153
O5—H5⋯O8^v^	0.84	2.03	2.863 (4)	169

Symmetry codes: (i) 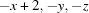; (ii) 
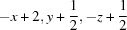
; (iii) 
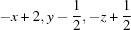
; (iv) 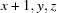; (v) 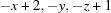.

**Fig. 1.**
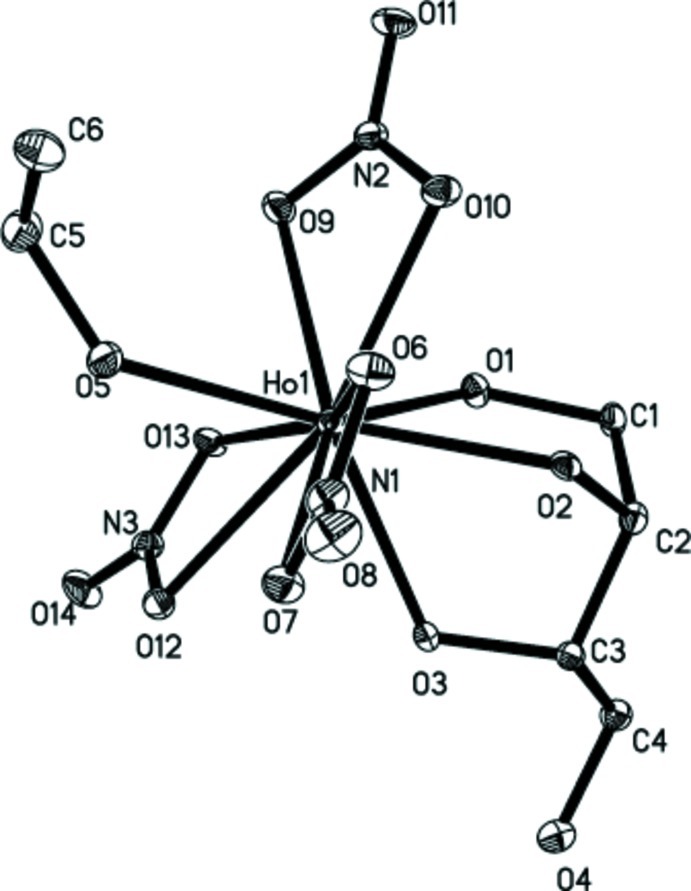
The molecular structure of the title complex, displacement ellipsoids drawn at 30% probability level. The Hydrogen atoms have been omitted for clarity.

## supplementary crystallographic information

### Comment

The interaction between carbohydrates and metal ions is of increasing interest as it occurs in many important biological processes (Gyurcsik & Nagy, 2000). Erythritol is a model compound to study the coordination behavior of hydroxyl groups to metal ions. For lanthanide nitrate-erythritol complexes, two kinds of metal complexes were observed: coordinate complex with water and coordinate complex without water (Yang *et al.*, 2003, 2004, 2012). The title holmium nitrate-erythritol complex is belonging to the complexes without water.

The title complex denoted as HoEN, where E stands for erythritol and N stands for nitrate, which is shown in Fig. 1. The coordinating number is 10 (three hydroxyl groups from one erythritol molecule, one hydroxyl group from ethanol, and three bidentate nitrate ions). Erythritol molecule is an O1,O2,O3-three hydroxyl group donor. The structure of HoEN is similar to NdEN, EuEN, YEN, GdEN and TbEN (Yang *et al.*, 2003, 2004, 2012). Because of the variation of ionic radii of rare earth elements, significant changes in Ln—O distances can be observed on comparison of LnEN complexes. Ho—O distances in the compound range from 2.364 to 2.590 Å, the average Ho—O distance is 2.444 Å. Y—O distances range from 2.358 to 2.594 Å, the average Y—O distance is 2.447 Å in YEN; Nd—O distances range from 2.455 to 2.620 Å, the average Nd—O distance is 2.528 Å in NdEN; Eu—O distances range from 2.421 to 2.600 Å, the average Eu—O distance is 2.494 Å in EuEN; Gd—O distances range from 2.398 to 2.596 Å, the average Gd—O distance is 2.478 Å in GdEN; Tb—O distances range from 2.373 to 2.581 Å, the average Tb—O distance is 2.410 Å in TbEN. The changes on M—O distances are consistent with the effect of lanthanide contraction.

The O-M-O (the oxygen atoms from coordinated hydroxyl groups of erythritol) bond angles are also variation of different LnEN complexes. O-Ho-O bond angles are 68.71 (9) (O1-Ho-O2), 68.54 (9) (O1-Ho-O3) and 64.58 (9)° (O2-Ho-O3). O-Y-O bond angles are 68.71 (12), 68.70 (11) and 64.32 (11)°. O-Nd-O bond angles are 66.57 (10), 66.54 (10) and 62.49 (10)°. O-Eu-O bond angles are 67.31 (8), 67.40 (8) and 63.62 (8)°. O-Gd-O bond angles are 67.97 (12), 67.66 (12)and 63.56 (12)°. O-Tb-O bond angles are 68.2 (3), 68.0 (3)and 63.7 (3)°. The changes of O-M-O bond angles are related with the changes of M—O distances.

### Experimental

Ho(NO_3_)_3_.6H_2_O (3 mmol) and erythritol (3 mmol) were dissolved in 6 ml water and 6 ml ethanol. The solution was put on a water bath, and the temperature was raised to 353 K. Small aliquots of EtOH were periodically added to the solution during the heating process to prolong the reaction time. The resulting mixtures were filtered and left for crystallization in room temperature, the suitable crystals for X-ray diffraction measurements were obtained in two weeks.

### Refinement

The C-bound H-atoms were placed in calculated positions (C—H = 0.93 Å) and were included in the refinement in the riding model approximation, U_iso_(H) = 1.2U_eq_(C). The O-bound H atoms were located in a difference Fourier map and were refined with distance restranits of O—H = 0.84 Å, U_iso_(H) = 1.2U_eq_(O).

### Crystal data

**Table:**

[Ho(NO_3_)_3_(C_4_H_10_O_4_)(C_2_H_6_O)]	*F*(000) = 1008
*M**_r_* = 519.15	*D*_x_ = 2.333 Mg m^−^^3^
Monoclinic, *P*2_1_/*c*	Mo *K*α radiation, λ = 0.71073 Å
Hall symbol: -P 2ybc	Cell parameters from 5233 reflections
*a* = 7.7501 (16) Å	θ = 2.1–27.5°
*b* = 12.783 (3) Å	µ = 5.44 mm^−^^1^
*c* = 15.164 (3) Å	*T* = 173 K
β = 100.35 (3)°	Block, colorless
*V* = 1477.8 (5) Å^3^	0.26 × 0.19 × 0.19 mm
*Z* = 4	

### Data collection

**Table:**

Rigaku Saturn724+ CCD diffractometer	3376 independent reflections
Radiation source: fine-focus sealed tube	3198 reflections with *I* > 2σ(*I*)
Graphite monochromator	*R*_int_ = 0.038
Detector resolution: 28.5714 pixels mm^-1^	θ_max_ = 27.5°, θ_min_ = 2.1°
ω scans at fixed χ = 45°	*h* = −10→9
Absorption correction: multi-scan (*CrystalClear*; Rigaku, 2007)	*k* = −14→16
*T*_min_ = 0.25, *T*_max_ = 0.36	*l* = −19→19
10146 measured reflections	

### Refinement

**Table:**

Refinement on *F*^2^	Primary atom site location: structure-invariant direct methods
Least-squares matrix: full	Secondary atom site location: difference Fourier map
*R*[*F*^2^ > 2σ(*F*^2^)] = 0.029	Hydrogen site location: inferred from neighbouring sites
*wR*(*F*^2^) = 0.061	H-atom parameters constrained
*S* = 1.19	*w* = 1/[σ^2^(*F*_o_^2^) + (0.0123*P*)^2^ + 3.0745*P*] where *P* = (*F*_o_^2^ + 2*F*_c_^2^)/3
3376 reflections	(Δ/σ)_max_ = 0.001
218 parameters	Δρ_max_ = 1.11 e Å^−^^3^
0 restraints	Δρ_min_ = −0.83 e Å^−^^3^

### Special details

**Table:**

Geometry. All esds (except the esd in the dihedral angle between two l.s. planes) are estimated using the full covariance matrix. The cell esds are taken into account individually in the estimation of esds in distances, angles and torsion angles; correlations between esds in cell parameters are only used when they are defined by crystal symmetry. An approximate (isotropic) treatment of cell esds is used for estimating esds involving l.s. planes.
Refinement. Refinement of F^2^ against ALL reflections. The weighted R-factor wR and goodness of fit S are based on F^2^, conventional R-factors R are based on F, with F set to zero for negative F^2^. The threshold expression of F^2^ > 2sigma(F^2^) is used only for calculating R-factors(gt) etc. and is not relevant to the choice of reflections for refinement. R-factors based on F^2^ are statistically about twice as large as those based on F, and R- factors based on ALL data will be even larger.

### Fractional atomic coordinates and isotropic or equivalent isotropic displacement parameters (Å^2^)

**Table:**

	*x*	*y*	*z*	*U*_iso_*/*U*_eq_	
Ho1	0.87485 (2)	0.104079 (12)	0.252907 (11)	0.01270 (6)	
O1	0.8663 (3)	0.1741 (2)	0.10772 (18)	0.0164 (6)	
H1	0.8036	0.1410	0.0660	0.025*	
O2	1.1423 (3)	0.1887 (2)	0.24123 (17)	0.0148 (6)	
H2	1.1406	0.2509	0.2589	0.022*	
O3	1.0696 (3)	0.00635 (19)	0.16970 (17)	0.0150 (5)	
H3	1.0969	−0.0551	0.1861	0.023*	
O4	1.2729 (4)	−0.0786 (2)	0.04468 (19)	0.0211 (6)	
H4	1.3661	−0.0846	0.0823	0.025*	
O5	0.6941 (4)	0.0507 (2)	0.3547 (2)	0.0226 (6)	
H5	0.7317	0.0052	0.3933	0.027*	
O6	1.0213 (4)	0.1747 (2)	0.39669 (19)	0.0231 (6)	
O7	1.0682 (4)	0.0119 (2)	0.3731 (2)	0.0229 (6)	
O8	1.2006 (4)	0.0879 (3)	0.4970 (2)	0.0307 (7)	
O9	0.6021 (4)	0.2037 (2)	0.2129 (2)	0.0213 (6)	
O10	0.8267 (4)	0.2959 (2)	0.2684 (2)	0.0209 (6)	
O11	0.5809 (4)	0.3735 (2)	0.2135 (2)	0.0281 (7)	
O12	0.8170 (4)	−0.0952 (2)	0.23689 (19)	0.0178 (6)	
O13	0.6491 (4)	0.0108 (2)	0.14891 (19)	0.0185 (6)	
O14	0.6096 (4)	−0.1565 (2)	0.1343 (2)	0.0279 (7)	
N1	1.0997 (5)	0.0912 (3)	0.4249 (2)	0.0200 (7)	
N2	0.6662 (4)	0.2943 (3)	0.2309 (2)	0.0178 (7)	
N3	0.6893 (4)	−0.0825 (3)	0.1723 (2)	0.0175 (7)	
C1	1.0234 (5)	0.2218 (3)	0.0869 (3)	0.0178 (8)	
H1A	1.0180	0.2987	0.0934	0.021*	
H1B	1.0350	0.2055	0.0244	0.021*	
C2	1.1784 (5)	0.1781 (3)	0.1517 (3)	0.0157 (8)	
H2A	1.2861	0.2190	0.1464	0.019*	
C3	1.2153 (5)	0.0621 (3)	0.1435 (3)	0.0144 (7)	
H3A	1.3245	0.0444	0.1869	0.017*	
C4	1.2410 (5)	0.0312 (3)	0.0504 (3)	0.0183 (8)	
H4A	1.1350	0.0501	0.0065	0.022*	
H4B	1.3415	0.0704	0.0348	0.022*	
C5	0.5492 (6)	0.0933 (3)	0.3922 (3)	0.0270 (10)	
H5A	0.4776	0.0351	0.4092	0.032*	
H5B	0.4735	0.1356	0.3460	0.032*	
C6	0.6115 (6)	0.1606 (3)	0.4734 (3)	0.0287 (10)	
H6A	0.6832	0.1184	0.5202	0.043*	
H6B	0.5100	0.1884	0.4961	0.043*	
H6C	0.6817	0.2187	0.4568	0.043*	

### Atomic displacement parameters (Å^2^)

**Table:**

	*U*^11^	*U*^22^	*U*^33^	*U*^12^	*U*^13^	*U*^23^
Ho1	0.01219 (10)	0.01246 (10)	0.01306 (10)	0.00054 (6)	0.00127 (7)	0.00034 (6)
O1	0.0137 (14)	0.0171 (14)	0.0166 (14)	−0.0037 (11)	−0.0024 (11)	0.0009 (10)
O2	0.0186 (14)	0.0102 (13)	0.0160 (14)	−0.0010 (11)	0.0043 (11)	−0.0037 (10)
O3	0.0164 (14)	0.0118 (13)	0.0178 (14)	−0.0010 (11)	0.0055 (11)	0.0020 (10)
O4	0.0194 (15)	0.0230 (15)	0.0199 (15)	0.0016 (12)	0.0005 (12)	−0.0056 (11)
O5	0.0223 (15)	0.0237 (16)	0.0232 (16)	0.0044 (12)	0.0080 (13)	0.0064 (12)
O6	0.0278 (17)	0.0220 (16)	0.0179 (15)	0.0052 (13)	−0.0001 (13)	−0.0004 (11)
O7	0.0230 (15)	0.0203 (15)	0.0243 (16)	0.0022 (12)	0.0017 (13)	0.0010 (12)
O8	0.0273 (18)	0.0436 (19)	0.0188 (16)	0.0073 (15)	−0.0024 (14)	0.0043 (13)
O9	0.0172 (14)	0.0150 (14)	0.0311 (17)	−0.0017 (12)	0.0026 (12)	0.0001 (12)
O10	0.0141 (14)	0.0198 (15)	0.0270 (16)	0.0023 (12)	−0.0013 (12)	−0.0045 (11)
O11	0.0257 (17)	0.0181 (15)	0.040 (2)	0.0102 (13)	0.0039 (15)	0.0035 (13)
O12	0.0122 (13)	0.0180 (14)	0.0219 (15)	0.0005 (11)	−0.0002 (12)	0.0033 (11)
O13	0.0169 (14)	0.0125 (13)	0.0243 (15)	0.0012 (11)	−0.0008 (12)	0.0017 (11)
O14	0.0308 (18)	0.0150 (15)	0.0356 (19)	−0.0063 (13)	−0.0003 (14)	−0.0042 (12)
N1	0.0182 (18)	0.0266 (19)	0.0147 (17)	0.0032 (15)	0.0019 (14)	0.0017 (14)
N2	0.0175 (17)	0.0168 (18)	0.0198 (17)	0.0038 (14)	0.0053 (14)	0.0012 (13)
N3	0.0160 (17)	0.0163 (17)	0.0201 (18)	0.0003 (14)	0.0033 (14)	−0.0019 (13)
C1	0.0162 (19)	0.018 (2)	0.020 (2)	−0.0032 (16)	0.0041 (16)	0.0033 (15)
C2	0.0152 (19)	0.0173 (19)	0.0153 (19)	−0.0023 (15)	0.0044 (15)	−0.0015 (14)
C3	0.0121 (18)	0.0134 (18)	0.0172 (19)	−0.0006 (15)	0.0010 (15)	−0.0001 (14)
C4	0.019 (2)	0.019 (2)	0.018 (2)	0.0010 (16)	0.0051 (16)	0.0007 (15)
C5	0.025 (2)	0.029 (2)	0.029 (2)	−0.0022 (19)	0.007 (2)	−0.0033 (18)
C6	0.033 (3)	0.024 (2)	0.028 (2)	0.0014 (19)	0.002 (2)	−0.0043 (18)

### Geometric parameters (Å, º)

**Table:**

Ho1—O1	2.367 (3)	O8—N1	1.225 (4)
Ho1—O2	2.373 (3)	O9—N2	1.271 (4)
Ho1—O3	2.473 (3)	O10—N2	1.272 (4)
Ho1—O5	2.364 (3)	O11—N2	1.212 (4)
Ho1—O6	2.443 (3)	O12—N3	1.272 (4)
Ho1—O7	2.444 (3)	O13—N3	1.267 (4)
Ho1—O9	2.449 (3)	O14—N3	1.217 (4)
Ho1—O10	2.497 (3)	C1—C2	1.515 (5)
Ho1—O12	2.590 (3)	C1—H1A	0.9900
Ho1—O13	2.445 (3)	C1—H1B	0.9900
O1—C1	1.446 (4)	C2—C3	1.520 (5)
O1—H1	0.8399	C2—H2A	1.0000
O2—C2	1.441 (4)	C3—C4	1.514 (5)
O2—H2	0.8400	C3—H3A	1.0000
O3—C3	1.450 (4)	C4—H4A	0.9900
O3—H3	0.8400	C4—H4B	0.9900
O4—C4	1.430 (5)	C5—C6	1.509 (6)
O4—H4	0.8401	C5—H5A	0.9900
O5—C5	1.453 (5)	C5—H5B	0.9900
O5—H5	0.8400	C6—H6A	0.9800
O6—N1	1.265 (4)	C6—H6B	0.9800
O7—N1	1.279 (4)	C6—H6C	0.9800
			
O5—Ho1—O1	142.74 (10)	C5—O5—Ho1	137.5 (2)
O5—Ho1—O2	143.95 (10)	C5—O5—H5	100.5
O1—Ho1—O2	68.71 (9)	Ho1—O5—H5	118.9
O5—Ho1—O6	76.03 (10)	N1—O6—Ho1	96.2 (2)
O1—Ho1—O6	128.42 (9)	N1—O7—Ho1	95.8 (2)
O2—Ho1—O6	68.05 (10)	N2—O9—Ho1	97.7 (2)
O5—Ho1—O7	74.33 (10)	N2—O10—Ho1	95.3 (2)
O1—Ho1—O7	141.90 (10)	N3—O12—Ho1	92.5 (2)
O2—Ho1—O7	81.29 (9)	N3—O13—Ho1	99.6 (2)
O6—Ho1—O7	52.28 (10)	O8—N1—O6	121.5 (3)
O5—Ho1—O13	80.85 (10)	O8—N1—O7	122.8 (3)
O1—Ho1—O13	71.78 (9)	O6—N1—O7	115.7 (3)
O2—Ho1—O13	135.05 (9)	O11—N2—O9	122.5 (3)
O6—Ho1—O13	156.88 (10)	O11—N2—O10	122.4 (3)
O7—Ho1—O13	121.18 (9)	O9—N2—O10	115.1 (3)
O5—Ho1—O9	74.06 (10)	O14—N3—O13	121.4 (3)
O1—Ho1—O9	72.19 (10)	O14—N3—O12	121.6 (3)
O2—Ho1—O9	118.12 (9)	O13—N3—O12	117.0 (3)
O6—Ho1—O9	105.73 (10)	O1—C1—C2	107.7 (3)
O7—Ho1—O9	145.26 (10)	O1—C1—H1A	110.2
O13—Ho1—O9	66.90 (9)	C2—C1—H1A	110.2
O5—Ho1—O3	132.46 (9)	O1—C1—H1B	110.2
O1—Ho1—O3	68.54 (9)	C2—C1—H1B	110.2
O2—Ho1—O3	64.58 (9)	H1A—C1—H1B	108.5
O6—Ho1—O3	114.44 (9)	O2—C2—C1	108.1 (3)
O7—Ho1—O3	77.77 (9)	O2—C2—C3	103.8 (3)
O13—Ho1—O3	81.74 (9)	C1—C2—C3	116.5 (3)
O9—Ho1—O3	135.65 (10)	O2—C2—H2A	109.4
O5—Ho1—O10	96.08 (10)	C1—C2—H2A	109.4
O1—Ho1—O10	74.71 (9)	C3—C2—H2A	109.4
O2—Ho1—O10	72.95 (9)	O3—C3—C4	111.5 (3)
O6—Ho1—O10	66.84 (9)	O3—C3—C2	106.9 (3)
O7—Ho1—O10	119.02 (9)	C4—C3—C2	112.9 (3)
O13—Ho1—O10	115.95 (9)	O3—C3—H3A	108.5
O9—Ho1—O10	51.40 (9)	C4—C3—H3A	108.5
O3—Ho1—O10	131.23 (9)	C2—C3—H3A	108.5
O5—Ho1—O12	70.39 (9)	O4—C4—C3	111.4 (3)
O1—Ho1—O12	108.02 (9)	O4—C4—H4A	109.3
O2—Ho1—O12	125.46 (9)	C3—C4—H4A	109.3
O6—Ho1—O12	119.47 (9)	O4—C4—H4B	109.3
O7—Ho1—O12	70.65 (9)	C3—C4—H4B	109.3
O13—Ho1—O12	50.82 (9)	H4A—C4—H4B	108.0
O9—Ho1—O12	111.14 (9)	O5—C5—C6	112.1 (4)
O3—Ho1—O12	64.32 (9)	O5—C5—H5A	109.2
O10—Ho1—O12	161.43 (9)	C6—C5—H5A	109.2
C1—O1—Ho1	118.6 (2)	O5—C5—H5B	109.2
C1—O1—H1	116.3	C6—C5—H5B	109.2
Ho1—O1—H1	115.3	H5A—C5—H5B	107.9
C2—O2—Ho1	110.3 (2)	C5—C6—H6A	109.5
C2—O2—H2	114.0	C5—C6—H6B	109.5
Ho1—O2—H2	110.1	H6A—C6—H6B	109.5
C3—O3—Ho1	117.9 (2)	C5—C6—H6C	109.5
C3—O3—H3	112.0	H6A—C6—H6C	109.5
Ho1—O3—H3	117.8	H6B—C6—H6C	109.5
C4—O4—H4	100.7		

### Hydrogen-bond geometry (Å, º)

**Table:**

*D*—H···*A*	*D*—H	H···*A*	*D*···*A*	*D*—H···*A*
O1—H1···O4^i^	0.84	1.86	2.665 (4)	161
O2—H2···O12^ii^	0.84	1.99	2.794 (4)	159
O3—H3···O10^iii^	0.84	2.08	2.914 (4)	177
O4—H4···O14^iv^	0.84	2.12	2.894 (4)	153
O5—H5···O8^v^	0.84	2.03	2.863 (4)	169

Symmetry codes: (i) −*x*+2, −*y*, −*z*; (ii) −*x*+2, *y*+1/2, −*z*+1/2; (iii) −*x*+2, *y*−1/2, −*z*+1/2; (iv) *x*+1, *y*, *z*; (v) −*x*+2, −*y*, −*z*+1.
